# Feasibility of a Nurse-Led Weekend Group Exercise Program for People after Stroke

**DOI:** 10.1155/2017/4574385

**Published:** 2017-01-24

**Authors:** Katharine Scrivener, Raymond Tourany, Mary McNamara-Holmes, Karl Schurr, Simone Dorsch, Catherine Dean

**Affiliations:** ^1^Department of Health Professions, Faculty of Medicine and Health Sciences, Macquarie University, Ground Floor, 75 Talavera Rd, Sydney, NSW 2109, Australia; ^2^Bankstown-Lidcombe Hospital, Locked Bag 1600, Bankstown, NSW 2200, Australia; ^3^StrokeEd, P.O. Box 3105, Regents Park, NSW 2143, Australia

## Abstract

*Background.* Additional physical activity including repetitive task practice can improve outcomes after stroke. The additional practice can be facilitated by therapists and family members or could also be delivered by nursing staff.* Objective.* To investigate the feasibility of a nurse-led weekend exercise program after stroke.* Participants.* Individuals after stroke, who participated in a weekend exercise program during their hospital admission.* Methods.* A retrospective audit of the number of referrals to and amount of exercise repetitions achieved in a nurse-led weekend exercise program was undertaken. The weekend exercise program occurs on each Saturday and Sunday for one hour. The repetitions of exercise completed during each class were documented by staff. An audit was conducted to ascertain the amount and type of exercise completed within the class.* Results.* During the study period 284 people were referred to the exercise program. The mean number of exercise repetitions completed per participant in each class was 180.7 (SD 205.4). The number of exercise repetitions completed by participants was highly variable ranging from 0 to 1190 per class.* Conclusion.* The amount of average exercise repetitions completed in the Weekend Warrior program was large but with significant variability. A nurse-led exercise class is a feasible method of delivering exercise opportunities to individuals in hospital after stroke.

## 1. Introduction

Observational studies have consistently shown that individuals in hospital after stroke spend large proportions of the waking day alone and inactive [1–3]. Moreover, during the limited daily active time, activities are normally of low intensity [3]. This lack of activity may result in a longer hospital stay and impact on functional outcomes.

In 2011, an observational study conducted at the site of this study demonstrated that individuals admitted to this stroke unit were particularly inactive during the weekend when therapy staff were not working and therapy areas were closed [4]. Significantly more time was spent in the bedroom on the weekend compared to during the week (97% compared to 76%) and more time was spent alone (64% compared to 43%). Moreover, significantly less time was spent completing therapeutic activities on weekend days compared with weekdays (5% compared to 15%) [4]. These results are comparable to those reported in a recent systematic review, demonstrating low activity levels on weekends in hospital after stroke [3].

Being physically active is important in the rehabilitation of movement problems after stroke [5, 6]. Physical activity may comprise various aspects such as decreasing sedentary time, performing general daily activities, and completing specific therapeutic activities. There are different benefits associated with the various forms of physical activity. For example, decreasing sedentary time may contribute to stroke prevention [7, 8] and therapeutic activities may improve an individual's ability to perform daily activities, participate within the community, and improve quality of life [5, 6, 9].

Numerous systematic reviews have demonstrated that more intensive therapeutic activities early after stroke, particularly repetitive task training, are associated with improved outcomes [6, 9–11]. More specifically, providing additional in-hospital rehabilitation after hours or on the weekend is effective in increasing physical activity and functional outcomes without any adverse consequences [12]. Despite this body of evidence, recent evidence suggests that completing additional repetitions of task training may not be effective at all time points after stroke and for all functional tasks [13]. Further research regarding the required dose of repetitive task training at various time points is needed.

To increase physical activity over the weekend on the stroke unit, the physiotherapy and nursing staff at Bankstown-Lidcombe Hospital designed and implemented a physiotherapist-prescribed and nurse-led weekend exercise program, called the Weekend Warriors. The Weekend Warrior program is a three-level program with each level comprising three exercises. The program focuses on lower limb exercises that emphasise leg strengthening and balance using repetitive task practice of sitting, standing up, and standing and stepping. The development of the exercise program was based on evidence regarding high intensity repetitive task practice [5, 6, 9] and that a group program is an effective way of delivering exercise to people after stroke [14].

The aim of this retrospective study was to determine the feasibility of a nurse-led weekend exercise program for people after stroke. To do this we retrospectively analysed the number of referrals to the program and the amount of exercise repetitions completed in the program at Bankstown-Lidcombe Hospital over a 17-month period (between June 2013 and November 2015). This allowed us to reflect on the number of people accessing the exercise program and the impact of the program on participants' physical activity over the weekend.

Data collected was compared to predetermined feasibility criteria. For the program to be deemed feasible,

1. referrals to the program averaged greater than five per week and were maintained over time,
2. exercise repetitions for each participant within a class averaged greater than 60,
3. minimal variability between exercise repetitions on Saturday and Sunday meant the program was successful in achieving its aim on both days,
4. minimal variability between exercise repetitions within each level of the program and mobility level meant the class was inclusive for participants of all levels of disability.

## 2. Methods

### 2.1. Design

A retrospective data audit was completed to summarise the number of referrals and physical activity (measured via exercise repetitions) completed in the Weekend Warrior program at Bankstown-Lidcombe Hospital between June 2013 and November 2015. Ethical approval was obtained for a retrospective study of existing data (HREC number HREC/14/LPOOL/258).

### 2.2. Weekend Warrior Program

The Weekend Warrior program is designed to increase physical activity on the weekend. The physiotherapists on the stroke unit select appropriate participants for the program and determine if any modifications of the exercises are needed to participate in the group exercise class. Participants are referred to the program if they are safely able to complete repetitive exercise in a semisupervised environment and have goals related to lower limb activities. There are no exclusion criteria for the program. Participants with cognitive or language impairments are included in the program if they can participate, with support of family or carers if applicable.

Two nursing staff members are rostered to conduct the class on each weekend day by the nursing unit manager. All nursing staff were included in the roster. The nurses assist participants to the dining room, where the class occurs, and then assist them to set up and complete the prescribed exercise program. Nursing staff have received training in exercise set-up, basic coaching, and procedures for counting exercise repetitions. They also received an instruction manual including information on how to set up the class and conduct each exercise. Senior nursing staff and management provided ongoing feedback and advice. During the hour class the remaining staff reallocate supervision of patients in the ward environment, including acute patients. Nursing and therapy staff met regularly to discuss the exercise program and resolve any challenges to implementation.

The Weekend Warrior program has three levels of difficulty to enable people of all abilities to attend. Level 1 can be completed in sitting; therefore, people who are limited to a wheelchair can complete this level. Levels 2 and 3 contain exercises in standing with progressive levels of difficulty. All exercises focus on lower limb strength and repetitive practice of functional tasks. The Weekend Warrior program is completed on both Saturday and Sunday for one hour each day.

Table 1 provides a summary of the exercises in the Weekend Warrior program.

### 2.3. Study Site

This study was conducted at Bankstown-Lidcombe Hospital. The hospital has a comprehensive stroke unit consisting of 20 beds. This unit is a colocated acute and rehabilitation unit which sees individuals after stroke through their hospital admission. The average length of stay on the unit in 2015 was 15.1 days. Stroke is the most common diagnosis for people on the unit; however, people with other neurological conditions and mixed diagnoses are occasionally admitted. The average age of individuals admitted to the unit is 76.2 years; half of admissions have cognitive impairments and score 1.7 on the Charlston Comorbidity index [15].

Inpatients complete usual therapy during the week, which commonly includes twice daily physiotherapy in the rehabilitation gym along with occupational and speech therapy. There is no formal therapeutic activity on the weekend and no therapy staff present and therapy areas are closed.

### 2.4. Participants

Individuals after stroke admitted to the stroke unit who during their admission participated in the Weekend Warrior exercise program.

### 2.5. Protocol

The physiotherapy and nursing staff on the Stroke Unit at Bankstown-Lidcombe Hospital have conducted the Weekend Warrior program since August 2011, with exercise repetition data being collected from June 2013.

During the exercise class each participant's exercise repetitions are counted by class participants, carers, or nursing staff using hand held tally counters. Nursing staff recorded this number on each participant's exercise recording sheet. This procedure has been previously validated at the study hospital [16].

The participant exercise recording sheet can be seen in Appendix. The total number of repetitions completed by the participant for each exercise on Saturday or Sunday was recorded in the appropriate box within this sheet.

The nursing staff collected the recording sheets after each Weekend Warrior class and they were given to the physiotherapy staff each Monday morning with a general report of the class and any issues that arose. The data sheets were then stored in a secure cabinet in the Physiotherapy Department.

### 2.6. Statistical Analysis

Data was entered into a Microsoft Excel spreadsheet with each exercise tabulated for each individual participant and for both Saturday and Sunday sessions. For data analysis SPSS Version 21 was used. The amount of exercise completed by each participant was analysed using descriptive statistics including mean, standard deviation, median, and interquartile range (IQR). Other participant information from the exercise recording sheets such as program level and physical assistance needed for mobility was also recorded.

The variation in amount of repetitions completed per participant between Saturday and Sunday exercise classes and between each program level was examined with an independent *t*-test.

## 3. Results

### 3.1. Flow of Participants through the Study

Between June 2013 and November 2015 there were 384 patients referred to the Weekend Warrior program. Many class participants were referred to the class over multiple weeks with the mean time in the class being 2.7 weeks (median 2 weeks, range 1 to 23). Twelve of the participants were referred to the class during more than one admission to the stroke unit. Fifty eight percent of participants were male.

There was a total of 1068 individual referrals with an average of 8.6 referrals for each class with a participant to staff ratio of 4.6 : 1. Exercise data was available for 709 of these referrals (66%). There were nine weeks in the study period for which there was no referral data.

Most participants were referred to program level 2 (40.5%). Level 1 (31.5%) and level 3 (28%) had similar rates of referrals. The participants' mobility varied with 9% reliant on a wheelchair, 41% needed physical assistance to transfer and walk, and 50% did not require physical assistance.

### 3.2. Total Exercise Repetitions per Participant within Each Class

The mean number of total exercise repetitions per participant in each class was 180.7 (SD 205.4). The range of exercise repetitions completed by each participant was 0 to 1190.

### 3.3. Total Number of Exercise Repetitions per Participant on Each Day

Table 2 summarises the total exercise repetitions per participant within each class, with Saturday and Sunday comparisons.

### 3.4. Total Number of Exercise Repetitions per Participant for Each Level of the Weekend Warrior Program

Level 3 contained the largest mean number of exercise repetitions per participant in each class with 208.7 exercise repetitions completed.  Table 3 summarises exercise repetitions in each program level.

There were significantly fewer exercise repetitions completed per participant in level 2 of the program compared to level 3 (mean difference −47.0, 95% CI −74.4 to −19.6, *p* < 0.01). There were no other significant differences between program levels.

### 3.5. Total Number of Exercise Repetitions per Participant for Each Level of Mobility

There were small differences in exercise repetitions between mobility levels that were not significant. Further details can be seen in Table 4.

### 3.6. Comparison of Study Results with Predetermined Feasibility Criteria

The study results demonstrate that the program met all aspects of the predetermined feasibility criteria. Please refer to Table 5 to see details regarding the comparison of results to the feasibility criteria.

## 4. Discussion

This study is the first retrospective study to demonstrate that a nurse-led exercise class completed in inpatient stroke rehabilitation is feasible. The program met the predetermined feasibility criteria. Firstly, a high number of referrals were maintained over a long period (29 months). Secondly, this study found that a large amount of exercise occurred in the program with an average of 190.6 (SD 237.1) exercise repetitions per class, well above the 60 decided by the authors. There was a high degree of variability of exercise repetitions completed. Some participants completed over 1000 repetitions within a single one-hour class meaning more than 15 exercise repetitions each minute for the hour, whilst other participants refused to trial any of the exercises and completed zero exercise repetitions.

There were no significant differences between the amount of exercise repetitions that occurred on either Saturday or Sunday. Furthermore, participants with better functional mobility did not complete more repetitions than participants with lower functional mobility despite being able to exercise more independently. This suggests that the graded program, with three levels of exercise difficulty, includes people with all levels of ability after stroke.

**Figure 1:**
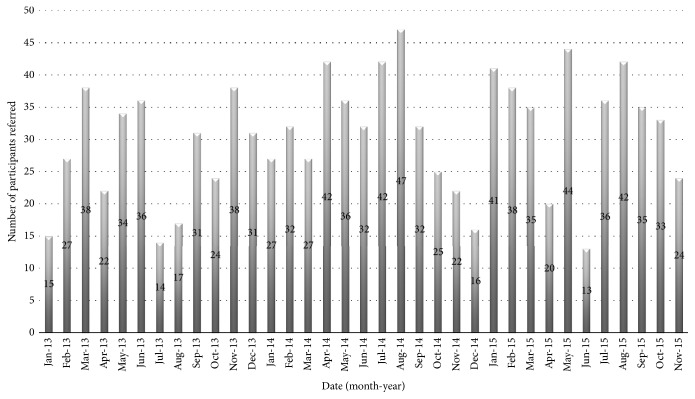
Number of participants referred to the program per month.

The implementation of the program had both achievements and challenges. The achievements such as referral rate and consistency can be attributed to a group of dedicated nursing and physiotherapy staff who drove the implementation of the program. The support from nursing management including training and rostering all nursing staff to conduct the program ensured that the program had longevity. The challenges included some nursing staff being reluctant to lead the program believing it was beyond the scope of his/her role. As seen in Figure 1, many referrals were received for the program and this was maintained over time. This is a credit to staff on the stroke unit.

Previous studies have recorded repetitions of exercise to quantify the intensity of physiotherapy and occupational therapy sessions after stroke [17–20]. In a study investigating outpatient physiotherapy and occupational therapy sessions a total of 362.4 exercise repetitions (stepping, balance, transfers, and active exercise) were completed per 36-minute session. This is a rate of approximately 10 repetitions a minute. A prospective cohort study conducted by Scrivener and colleagues in 2012 investigated exercise dose and mobility outcome on the Bankstown-Lidcombe Stroke Unit [15]. This study found that individuals after stroke completed an average of 288 (SD 242) exercise repetitions of lower limb exercises in physiotherapy each day and were capable of up to 1136 exercise repetitions per day. Furthermore, it has been shown that 300 repetitions of upper limb exercise are possible in a one-hour exercise class [21]. In the current study a comparable average of 181 repetitions was achieved in a one-hour nurse-led class.

The current study demonstrated the feasibility of conducting a nurse-led weekend exercise program for people in hospital after stroke. Future studies are needed to determine if the program can improve functional outcomes. A study investigating the provision of additional one-to-one nursing to practice functional activities on the weekend [21] showed no improvements in patient outcome. This can possibly be explained by the low intensity of the intervention with an average of only 13 (SD 14) additional minutes of nurse-led task practice on each weekend day [21]. Other studies have investigated providing additional exercise programs that are self-administered or supervised by family members [22, 23]. Encouragingly, these studies have demonstrated a positive effect on balance [22] and upper limb function [23]. In these studies, the increased intensity of practice of upper and lower limb functional tasks has had positive impact on outcome.

The strength of this study is the long time-period (29 months) over which data collection occurred. However, there are several study limitations. Firstly, it was a retrospective study and the data that was collected was limited to the data that had been recorded on the existing data sheets. For example, we had hoped to report further demographic information regarding the class participants; however, this was not routinely collected. Furthermore, there was a large amount of data missing with 34% of referrals to the class having no exercise data. A large proportion of these participants would have no exercise data because they did not participate in the Weekend Warrior program for a variety of reasons. Unfortunately, this assumption cannot be verified as some data sheets may have been lost and not correctly handed over or filed.

In conclusion, the implementation of a nurse-led weekend exercise program after stroke was feasible with many referrals to the program and a high average amount of exercise completed by participants. Moreover, the program successfully catered for participants at all levels of disability. However, the amount of exercise recorded was variable with large discrepancies between minimum and maximum amounts of repetitions completed.

## Figures and Tables

**Figure 2:**
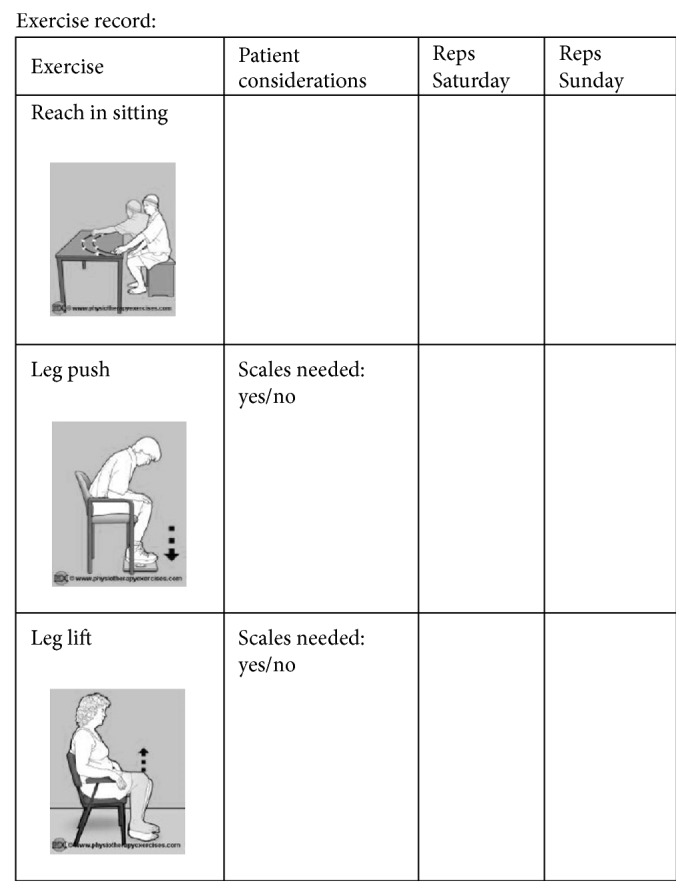
Images copied with permission of http://www.physiotherapyexercises.com, a freely available website for exercise prescription.

**Table 1:** Details of the Weekend Warrior exercise program.

Program level	Exercises
Level 1	*Exercise 1*. Leg push: in sitting, the participant is instructed to push down through the heel of their affected leg and hold for 5 seconds. Scales are commonly placed under the foot to provide concurrent feedback about the size of the push generated. The participant aims to complete 50 repetitions. *Exercise 2*. Leg lift: in sitting, the participant lifts their affected leg (by flexing at the hip) while keeping the knee flexed at 90 degrees. The participant aims to complete 30 repetitions. *Exercise 3*. Sitting balance: the participant reaches for a cup with their unaffected arm with the cup positioned 5 cm greater than the length of the participant's arm. There is an emphasis on the participant pushing through their legs to control their movement forward. The participant aims to complete 50 repetitions.

Level 2	*Exercise 1*. Sitting balance: as per exercise 3 above. *Exercise 2*. Sit-to-stand: participant starts in sitting with the chair at a height recommended by the referring physiotherapist. They then stand up without the use of hands (if possible). The participant is instructed to stand up straight. The participant aims to complete 50 repetitions. *Exercise 3*. Standing balance: the participant stands near a table. An object is placed on a table (arm's length away). The object is then picked up from one side and moved to the other side (using unaffected arm). Object is moved from side to side to transfer the weight from one leg to the other. The participant aims to complete 100 repetitions.

Level 3	*Exercise 1*. Sit-to-stand: as per exercise 2 above. *Exercise 2*. Standing balance: as per exercise 3 above. *Exercise 3*. Stepping: participant is instructed to take a step forward with unaffected leg and then step unaffected leg backwards so feet are level. Use of a block is optional. They then step forward with affected leg. The participant aims to complete 50 repetitions.

**Table 2:** Amount of exercise repetitions completed in each Weekend Warrior class.

	Mean (SD)	Median (IQR)	Range	Difference between days (Saturday minus Sunday)
Total exercise repetitions *n* = 1113	180.7 (205.4)	127 (280)	0 to 1190	
Saturday *n* = 568	185.8 (203.7)	145.5 (285)	0 to 1150	10.4 (−13.8 to 34.6)
Sunday *n* = 545	175.4 (207.2)	110 (265)	0 to 1190	

**Table 3:** Comparison of the amount of exercise repetitions completed in each Weekend Warrior class for program level.

	Mean (SD)	Median (IQR)	Range	Difference between program levels
Level 1 *n* = 360	180.5 (235.9)	93.5 (269)	0 to 1190	Compared to level 2: 18.8 (95% CI −9.6 to 47.1) Compared to level 3: −28.3 (95% CI −62.3 to 5.7)
Level 2 *n* = 448	161.7 (175.0)	123.5 (250)	0 to 1175	Compared to level 1: see above Compared to level 3: −47.0 (95% CI −74.4 to −19.6)
Level 3 *n* = 305	208.7 (205.7)	180 (290)	0 to 1106	See above

**Table 4:** Comparison of the amount of exercise repetitions completed in each Weekend Warrior class for mobility level.

Mobility	Mean (SD)	Median (IQR)	Range	Difference between program levels
No assistance *n* = 497	186.6 (193.9)	150 (299)	0 to 1150	Compared to physical assistance: 12.4 (95% CI −13.8 to 38.7) Compared to hoist/wheelchair: 23.6 (95% CI −24.7 to 71.9)
Physical assistance *n* = 438	174.2 (215.9)	104 (255)	0 to 1175	Compared to no assistance: see above Compared to hoist/wheelchair: 11.1 (95% CI −42.2 to 64.5)
Hoist/wheelchair *n* = 76	163.0 (234.4)	77.5 (215)	0 to 1110	See above

**Table 5:** Comparison of the study results to the predetermined feasibility criteria.

Predetermined feasibility criteria	Study results
(1) Referrals to the program averaged greater than 5 a week and were maintained over time.	(1) Referrals to the program averaged 8.6 per week. Figure 1 demonstrates that the referrals were maintained over time.

(2) Exercise repetitions for each participant within a class averaged greater than 60.	(2) Exercise repetitions for each participant within a class averaged 180.7.

(3) There was minimal variability between exercise repetitions on Saturday and Sunday, meaning the program was successful in achieving its aim on both days.	(3) There was no significant difference between exercise repetitions completed on Saturday and Sunday (mean difference 10.4, 95% CI −13.8 to 34.6).

(4) There was minimal variability between exercise repetitions within each level of the program and mobility level meaning the class was inclusive for participants of all levels of disability.	(4) There were 47 fewer exercise repetitions completed per participant in level 2 of the program compared to level 3 (95% CI −74.4 to −19.6, *p* < 0.01). There were no other significant differences in exercise repetitions between program and mobility levels.

## Appendix

### Abbreviated Exercise Recording Sheet

See Figure 2.
